# Statistical analysis plan for the Alveolar Recruitment for Acute Respiratory Distress Syndrome Trial (ART). A randomized controlled trial

**DOI:** 10.5935/0103-507X.20170024

**Published:** 2017

**Authors:** Lucas Petri Damiani, Otavio Berwanger, Denise Paisani, Ligia Nasi Laranjeira, Erica Aranha Suzumura, Marcelo Britto Passos Amato, Carlos Roberto Ribeiro Carvalho, Alexandre Biasi Cavalcanti

**Affiliations:** 1 HCor Research Institute - São Paulo (SP), Brasil.; 2 Divisão de Pneumologia, Instituto do Coração, Hospital das Clínicas, Faculdade de Medicina, Universidade de São Paulo - São Paulo (SP), Brasil.

**Keywords:** Acute respiratory distress syndrome, Positive-pressure respiration, Critically ill

## Abstract

**Background:**

The Alveolar Recruitment for Acute Respiratory Distress Syndrome Trial (ART) is an international multicenter randomized pragmatic controlled trial with allocation concealment involving 120 intensive care units in Brazil, Argentina, Colombia, Italy, Poland, Portugal, Malaysia, Spain, and Uruguay. The primary objective of ART is to determine whether maximum stepwise alveolar recruitment associated with PEEP titration, adjusted according to the static compliance of the respiratory system (ART strategy), is able to increase 28-day survival in patients with acute respiratory distress syndrome compared to conventional treatment (ARDSNet strategy).

**Objective:**

To describe the data management process and statistical analysis plan.

**Methods:**

The statistical analysis plan was designed by the trial executive committee and reviewed and approved by the trial steering committee. We provide an overview of the trial design with a special focus on describing the primary (28-day survival) and secondary outcomes. We describe our data management process, data monitoring committee, interim analyses, and sample size calculation. We describe our planned statistical analyses for primary and secondary outcomes as well as pre-specified subgroup analyses. We also provide details for presenting results, including mock tables for baseline characteristics, adherence to the protocol and effect on clinical outcomes.

**Conclusion:**

According to best trial practice, we report our statistical analysis plan and data management plan prior to locking the database and beginning analyses. We anticipate that this document will prevent analysis bias and enhance the utility of the reported results.

**Trial registration:**

ClinicalTrials.gov number, NCT01374022.

## Abstract (pt)

**Fundamentação:**

O estudo *Alveolar Recruitment for Acute Respiratory Distress Syndrome Trial* (ART) é um ensaio clínico internacional, multicêntrico, randomizado, pragmático e controlado com ocultação da alocação que envolve 120 unidades de terapia intensiva no Brasil, Argentina, Colômbia, Espanha, Itália, Polônia, Portugal, Malásia e Uruguai, com o objetivo primário de determinar se o recrutamento alveolar gradual máximo associado com titulação da pressão positiva expiratória final, ajustada segundo a complacência estática do sistema respiratório (estratégia ART), é capaz de aumentar, quando comparada aos resultados do tratamento convencional (estratégia ARDSNet), a sobrevivência em 28 dias de pacientes com síndrome do desconforto respiratório agudo.

**Objetivo:**

Descrever o processo de gerenciamento dos dados e o plano de análise estatística em um ensaio clínico internacional.

**Métodos:**

O plano de análise estatística foi delineado pelo comitê executivo e revisado pelo comitê diretivo do ART. Foi oferecida uma visão geral do delineamento do estudo, com foco especial na descrição de desfechos primário (sobrevivência aos 28 dias) e secundários. Foram descritos o processo de gerenciamento dos dados, o comitê de monitoramento de dados, a análise interina e o cálculo do tamanho da amostra. Também foram registrados o plano de análise estatística para os desfechos primário e secundários, e os subgrupos de análise pré-especificados. Detalhes para apresentação dos resultados, inclusive modelos de tabelas para as características basais, adesão ao protocolo e efeito nos desfechos clínicos, foram fornecidos.

**Conclusão:**

Em acordo com as melhores práticas em ensaios clínicos, submetemos nossos planos de análise estatística e de gerenciamento de dados para publicação antes do fechamento da base de dados e início das análises. Antecipamos que este documento deve prevenir viés em análises e incrementar a utilidade dos resultados a serem relatados.

**Registro do estudo:**

Número no registro ClinicalTrials.gov NCT01374022.

## INTRODUCTION

Alveolar collapse with reduction of functional lung size ("baby lung") is a hallmark of acute respiratory distress syndrome (ARDS).^(1)^ Although mechanical ventilation is needed to support life in patients with moderate-to-severe ARDS, it may damage lungs via two mechanisms: (1) overdistention and (2) cyclic opening and closing of small airways and alveoli (atelectrauma).^(2)^ Mechanical ventilation with low tidal volumes and low positive end-expiratory pressure (PEEP) decreases but does not eliminate ventilator-induced lung injury (VILI).^(3,4)^ Cyclic opening and closing of lung units persists with this strategy.^(5)^ The aim of recruitment maneuvers and PEEP titration is to open collapsed units and keep them open, thus minimizing atelectrauma and possibly dynamic overdistention.^(6)^ Most patients with ARDS for less than 72 hours are highly responsive to recruitment maneuvers, and serious adverse events are uncommon.^(7,8)^ However, the effect of recruitment maneuvers and PEEP titration on the clinical outcome of ARDS patients is uncertain. A systematic review with a meta-analysis of studies assessing recruitment maneuvers suggested a reduction in mortality; however, the quality of evidence is limited due to the high risk of bias in most primary studies and variable use of co-interventions, such as PEEP titration.^(9)^

The Alveolar Recruitment for Acute Respiratory Distress Syndrome Trial (ART) is an international multicenter randomized controlled trial that compares a strategy for maximum lung recruitment associated with PEEP titration adjusted according to the static compliance of the respiratory system to a conventional approach (ARDSNet protocol) for patients with moderate-severe ARDS.

This article outlines the statistical analysis plan for ART with the aim of preventing statistical analysis bias arising from exploratory analyses after the study results are known. The statistical analysis plan was developed prior to locking the trial database and starting analyses.

The primary objective of this study is to determine whether alveolar recruitment associated with PEEP titration adjusted according to the static compliance of the respiratory system (ART strategy) increases the 28-day survival rate of patients with moderate to severe ARDS compared to conventional treatment (ARDSNet strategy).

## METHODS

ART is an international multicenter randomized pragmatic controlled trial with allocation concealment and intention-to-treat analysis that compares a strategy of maximum lung recruitment associated with PEEP titration adjusted according to the static compliance of the respiratory system (ART strategy) to the ARDSNet approach for patients with moderate to severe ARDS. The trial is being conducted in 120 intensive care units in Brazil, Argentina, Colombia, Italy, Poland, Portugal, Malaysia, Spain, and Uruguay. The trial protocol was previously published and is registered with ClinicalTrials.gov (NCT01374022)^(10)^ and was approved by the Ethics Committee of all of the participant institutions.

Eligibility is evaluated in two phases, a screening phase and defining eligibility phase. In the screening phase, patients are considered for inclusion in the study if they are receiving invasive mechanical ventilation and have ARDS of less than 72 hours' duration. All of the following criteria should be met: acute onset respiratory failure; bilateral pulmonary infiltrate on chest X ray that is compatible with pulmonary edema; severe hypoxemia, defined as a partial pressure of arterial oxygen to fractional inspired oxygen ratio (PaO_2_/FIO_2_) ≤ 200 in arterial blood gases for less than 72 hours; absence of left atrial hypertension based on the medical team's evaluation (clinical or echocardiographic signs); and presence of a risk factor for lung injury. The exclusion criteria (exclusion if anyone is present) are as follows: age < 18 years; use of vasoconstrictor drugs in increasing doses over the past 2 hours (≥ 0.2mcg/kg per min for norepinephrine or ≥ 5mcg/kg per min for dopamine) or a mean arterial pressure < 65mmHg; contraindication of hypercapnia with intracranial hypertension or acute coronary syndrome; pneumothorax, subcutaneous emphysema, pneumomediastinum or pneumatocele; patient with no therapeutic perspective; candidate for palliative care exclusively (e.g., patient with imminent death, in moribund state or dying from cancer under exclusive palliative care); and previously randomized in the study.

While waiting for consent from a legal representative, we suggest ventilating the patient using a conventional approach (ARDSNet) as follows: volume-controlled mode, tidal volume of 4 - 6mL/kg of predicted body weight to ensure a plateau pressure ≤ 30cmH_2_O, PEEP and fractional inspired oxygen (FIO_2_) adjusted according to the ARDSNet table (Table 1) to maintain peripheral oxygen saturation (SpO_2_) ≥ 88% and arterial oxygen partial pressure (PaO_2_) ≥ 55mmHg, flow of 60L/min (may be reduced if peak pressure > 45cmH_2_O), descending waveform, inspiratory to expiratory ratio (I:E) of 1:1 to 1:2, inspiratory pause of 0.5 second (may be reduced if I:E ratio is inverted), and respiratory rate to maintain the partial pressure of carbon dioxide (PaCO_2_) between 35mmHg and 60mmHg. Alveolar recruitment maneuvers should be avoided.

**Table 1:** ARDSNet table of the fraction of inspired oxygen and positive end-expiratory pressure values to maintain peripheral oxygen saturation ≥ 88% and partial pressure of arterial oxygen ≥ 55mmHg

**FIO_2_ (%)**	30	40	40	50	50	60	70	70	70	80	90	90	90	100
**PEEP**	5	5	8	8	10	10	10	12	14	14	14	16	18	18-24

FIO_2_ - fraction of inspired oxygen; PEEP - positive end-expiratory pressure.

After three hours of mechanical ventilation according to the ARDSNet protocol, FIO_2_ is adjusted to 100% and PEEP to 10cmH_2_O (except if PEEP was previously ≥ 16cmH_2_O; in this case PEEP is maintained) for 30 minutes, after which the arterial blood gases are measured. Patients are considered eligible if the PaO_2_ measured with FIO_2_ = 100% and PEEP = 10cmH_2_O (or ≥ 16cmH_2_O) is 200mmHg or less, and less than 72 hours have been spent since the first time PaO_2_/FIO_2_ ≤ 200 was determined.

### Randomization

Eligible patients are randomly allocated in a 1:1 ratio for treatment with either the ART or ARDSNet strategy. The randomization list is generated electronically using appropriate software. Randomization is performed in blocks with stratification by center, age (≤ 55 or > 55 years-old) and PaO_2_/FIO_2_ ratio (≤ 100 or > 100mmHg).

Allocation concealment is maintained by means of a web-based central automated randomization system that is available 24 hours a day (ACT-Clinic) and was developed by a team of programmers and investigators from the Research Institute HCor. The group to which the patient is allocated is disclosed only after the patient is registered in the electronic system. This prevents the investigator and medical team from predicting the treatment group to which the patient will be allocated. To include a patient in the study, investigators must simply access the HCor Data Management System website (https://servicos.hcor.com.br/iep/estudoclinico) and fill out a short medical record form.

### Treatment groups

**Figura 1:**
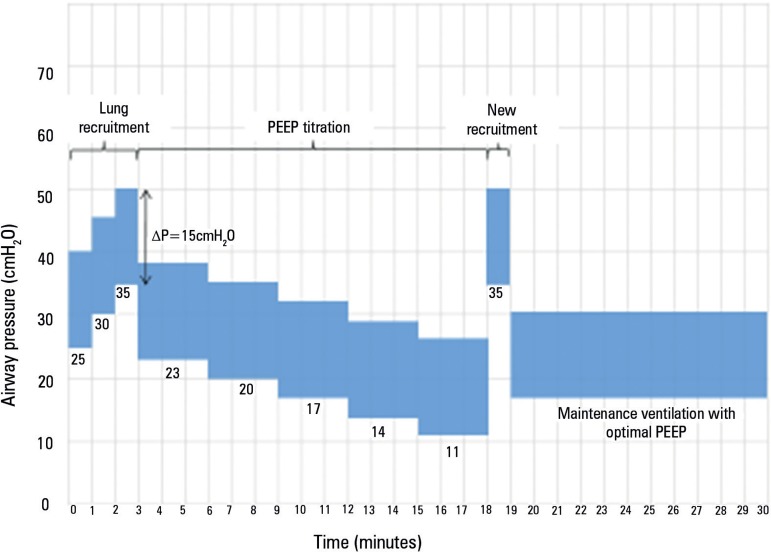
Schematic representation of the Alveolar Recruitment for ARDS Trial strategy with the recruitment maneuver and positive end-expiratory pressure titration according to the static compliance of the respiratory system.

Patients randomly assigned to the ART group undergo alveolar recruitment with incremental PEEP levels, followed by PEEP titration according to the static compliance of the respiratory system and a new recruitment. After recruitment and PEEP titration, patients are ventilated in controlled volume mode with PEEP set at the titrated value for at least 24 hours. Figure 1 shows a schematic representation of the recruitment maneuver followed by PEEP titration.

The recruitment maneuver and PEEP (positive end-expiratory pressure) titration are initiated only after a protocolized preparation that included: (1) sedation and neuromuscular blockade; (2) maintaining patients in the supine or prone position; (3) aspirating lower airway secretions; (4) installing a closed tracheal suctioning system as well as a heat and moisture exchanger; (5) assuring adequate monitoring, including and invasive blood pressure measurement; (6) correcting hypovolemia; (7) keeping the mean arterial pressure 75mmHg (if needed by starting or increasing vasopressors); (8) adjusting the respiratory rate to 35 breaths per minute for at least 20 minutes before recruitment; (9) disabling back-up or apnea ventilation. The recruitment maneuver is conducted in controlled pressure mode with a respiratory rate of 15 breaths per minute, FIO_2_=100%, and inspiratory to expiratory (I:E) ratio of 1:1. PEEP is set at 25cmH_2_O, with a pressure above PEEP of 15cmH_2_O for 1 minute. Then, PEEP is increased to 30cmH_2_O for 1 minute and finally to 35 cmH_2_O. After recruitment, PEEP titration is started with the following settings: a PEEP of 23cmH_2_O, volume controlled mode, tidal volume of 5mL/kg of predicted body weight, respiratory rate of 20 breaths per minute, flow of 30 L/min (square wave flow) and FIO_2_=100%. After 3 minutes, the static compliance of the respiratory system is calculated (with an inspiratory pause of 2 seconds). Then, PEEP is reduced by 3cmH_2_Oand maintained for 3 minutes, static compliance is measured again, and the steps are repeated until a PEEP of 11cmH_2_Ois reached. The ideal PEEP is the PEEP with the best static compliance of the respiratory system plus 2cmH_2_O. After PEEP titration, a new recruitment maneuver is conducted as follows: pressure-controlled mode, respiratory rate of 15 breaths per minute, FIO_2_=100%, inspiratory to expiratory (I:E) ratio of 1:1 and PEEP of 35cmH_2_O with a pressure above PEEP of 15cmH_2_O for 1 minute. Maintenance ventilation with optimal PEEP is started soon after this last recruitment maneuver.

In the first version of the protocol, we applied a recruitment maneuver using pressure controlled ventilation and a driving pressure of 15cmH_2_O. We started with a PEEP of 25cmH_2_O for 1 minute, followed by a PEEP of 35cmH_2_O (for 1 minute) and 45cmH_2_O (for 2 minutes). After recruitment, decremental PEEP titration was started with a PEEP of 23cmH_2_O in volume controlled mode and a tidal volume of 5mL/kg of predicted body weight. PEEP was decreased in steps of 3cmH_2_O to a minimum of 11cmH_2_O. After 4 minutes in each step, we measured the static compliance of the respiratory system. The PEEP associated with the best static compliance of the respiratory system plus 2cmH_2_O was considered to be the optimal PEEP. After PEEP titration, new recruitment with pressure controlled ventilation was carried out in one step using a PEEP of 45mH_2_O for 2 minutes. Then, maintenance ventilation was started in controlled volume mode with a tidal volume of 6mL/kg, using the optimal PEEP. The tidal volume was decreased to 5mL/kg or 4mL/kg if the plateau pressure exceeded 30cmH_2_O.

The steering committee proposed an amendment to the protocol after 3 cases of resuscitated cardiac arrest occurred in the experimental arm. The investigators considered that two of the adverse events were likely caused by respiratory acidosis and one by hemodynamic collapse, all possibly related to study interventions (recruitment maneuver and PEEP titration). The amendment was aimed at decreasing the risk of respiratory acidosis and hemodynamic impact of the recruitment maneuver. The amendment involved the following modifications to the experimental group protocol: (1) During recruitment, PEEP starts at 25cmH_2_O, then 30cmH_2_O and finally 35cmH_2_O. The maximum airway pressure reaches 50cmH_2_O (instead of 60cmH_2_O); (2) all of the recruitment steps last for 1 minute, totaling 3 minutes; (3) the PEEP titration steps are shortened to 3 minutes; and (4) after PEEP titration, recruitment is repeated with a PEEP of 35cmH_2_O for 1 minute. The steering committee consulted the Data Monitoring Committee, which agreed with the proposal. The amendment was implemented in June 18, 2015, starting with the 556^th^ patient enrolled in ART.

### Outcomes

Our primary outcome is 28-day survival.

Our secondary outcomes are: the length of intensive care unit (ICU) stay and hospitalization; ventilator-free days from day 1 until day 28; pneumothorax requiring drainage within 7 days; barotrauma within 7 days; and ICU, in-hospital and 6-month survival.

We consider pneumothorax to be any case requiring a chest tube within 7 days that is possibly due to barotrauma; that is, we do not consider cases judged to be clearly caused by invasive procedures, such as a central venous puncture or thoracocentesis, to be pneumothorax. We consider barotrauma to be any case within 7 days that displays any pneumothorax, pneumomediastinum, subcutaneous emphysema or pneumatocele > 2cm detected on image exams between randomization and 7 days, except those judged to be clearly caused by invasive procedures.

The trial also has some exploratory outcomes: death with refractory hypoxemia within 7 days (defined as PaO_2_ < 55mmHg in the last arterial blood gas analysis with FIO_2_ = 100%); death with refractory acidosis within 7 days (defined as pH ≤ 7.10 in the last arterial blood gas analysis); death with barotrauma within 7 days; cardiorespiratory arrest (defined as unexpected cardiac arrest, not due to progressive refractory shock) on day 1; need for commencement/increase of vasopressors or hypotension (mean arterial pressure < 65mmHg) within 1 hour after randomization; refractory hypoxemia (PaO_2_ < 55mmHg) within 1h after randomization; and severe acidosis (pH < 7.10) within 1h after randomization.

### Data management

The objective of our clinical data management plan is to provide high-quality data by adopting standardized procedures to minimize the number of errors and missing data, and consequently, to generate an accurate database for analysis.

### Responsibilities

The principal investigator at each center leads and/or supervises the daily operation of the project at his/her participating center and may appoint a Co-investigator and Research Coordinator. Most tasks can be delegated by the Principal Investigator to research professionals at the Participating Center provided that the professionals are qualified for such tasks and that the delegation is clearly recorded with the name of the professional and their role. However, the principal investigator is legally responsible for the study. The principal investigator is responsible for ensuring that the data are properly collected and entered into the Study Data Management System.

The Research Institute HCor assigns a coordinating team that includes a qualified data manager who is responsible for guaranteeing the data's accuracy during the process of data collection and analysis.

### Data collection

Data collection is performed using electronic case report forms via the Internet at the HCor Data Management System. The system has the following functions: patient registration, 24-hour randomization with allocation concealment, data input, data cleaning, and data export for statistical analysis. Data are entered directly into the system by each center team. All forms are electronically signed by the Principal Investigator of each center or by other appointed persons. Instructions for using the system will be made available to investigators.

### Quality assurance

Several strategies are performed to generate completeness and correctness of the clinical data. Investigators attended a training session before the start of the study to standardize procedures, including data collection. Study support material is available at all sites, and the investigators may contact the Study Coordinating Center to solve issues or problems that may arise.

Several problems can be detected by the system at the time of data entry. Subsequently, data monitoring is performed by a data management team in the central office that looks for missing data and inconsistencies using routines implemented in R software. In this sense, missing, inconsistent, illogical, out of range and discrepant data will be marked, and the participating sites will be notified for corrections or justifications. Weekly reports listing incomplete follow-up data and inconsistencies are referred to the sites. Resolution of queries by the investigator is updated in the database. If the investigator cannot provide a resolution, the reasons are collected in a spreadsheet. Finally, HCor staff contact all patients discharged alive from the hospital or their relatives to ensure that the reported 6-month follow-up vital status is accurate.

The data management team is also responsible for helping to detect cases of protocol deviation. When these situations occur, we program new training sessions at the site to revise the protocol. In addition, the data manager provides prospective reminders and protocol summaries by email regarding queries that are frequently detected.

### Database locking

The database will be locked as soon as all data are entered and all discrepant or missing data are resolved in the database or if all efforts are employed and we consider that the remaining issues cannot be fixed. At this step, our statisticians will review the data before database locking. We will fill out a database lock checklist before locking the database to ensure the completion of activities. After that, the study database will be locked and exported for statistical analysis. At this stage, permission to access the study database will be removed and the database will be archived.

### Storage and backup

Electronic files are archived in the HCor Server in a secure and controlled environment to maintain confidentiality. Electronic documents are controlled with password protection according to best practices.

### Trial organization and funding

The Research Institute Hcor is the sponsor and coordinator of the study. The Research Institute Hcor is primarily responsible for generating the randomization scheme and study database as well as for performing data quality assurance and data analysis. The trial structure includes the following groups: the coordinating center, the investigators, a steering committee and a data monitoring committee. The trial is endorsed by the Brazilian Research in Intensive Care Network (BRICNet).

The trial also receives institutional support from the Brazilian Association of Intensive Care Medicine (*Associação de Medicina Intensiva Brasileira* - AMIB) by means of its research network, AMIBNet.

The study is conducted as part of and funded by the Program to Support Institutional Development of the Universal Health System (PROADI-SUS) from the Brazilian Ministry of Health. The funding sources have no role in the design, execution, analysis, or decision to publish the results.

### Data monitoring committee and interim analyses

A Data Monitoring Committee (DMC) was established that included an independent epidemiologist, intensivist, and statistician in 2012 soon after the trial started. The responsibilities of the DMC are first to help ensure the safety of patients in the trial by protecting them from avoidable harm. Second, DMC provides the Steering Committee with advice about the conduct of the trial and integrity of the data to protect the validity and scientific credibility of the trial. However, the role of the DMC is limited on this issue because their detailed review of the progress of the trial only occurs infrequently. Third, the DMC evaluates interim analyses and judges efficacy, harm, and the net clinical effect.

Interim analyses to evaluate primary and secondary endpoints were conducted by an independent statistician and sent to the DMC after recruitment of approximately 33% and 66% of the sample, that is, when 172 and 344 deaths within 28 days had occurred. Based on these interim analyses, and possibly on external evidence, the DMC decided whether there was evidence beyond a reasonable doubt that the treatment was clearly contraindicated in all patients or any subgroup. The criterion for evidence beyond a reasonable doubt was increased mortality at 28 days with the maximum lung recruitment strategy compared with the low PEEP strategy, p < 0.01. Otherwise, the steering committee and other investigators were not informed of the results of the interim analyses. The two interim analyses were conducted, and the DMC recommended that the trial continue.

Considering previous evidence showing that: (1) early discontinuation of randomized trials due to benefits tends to produce biased estimates of effect (overestimation of the true effect), leading to erroneous medical guidelines and decisions; (2) according to the ethical principle of non-maleficence, a new treatment should not be used until there is clear objective evidence that it is beneficial; and (3) clinical practice usually does not change unless there is fairly convincing evidence of the advantages of a new treatment, which would be undermined if the study is discontinued early due to benefits, early discontinuation of an experimental treatment due to benefits may not be advantageous for future patients or may contribute to misleading guidelines. For these reasons, early discontinuation of the study due to the benefits of the experimental treatment was not planned.

Apart from conducting interim analyses of the primary and secondary outcomes, the DMC also received periodic reports (after multiples of 100 patients were enrolled) on the incidence of the following study adverse events: (1) need to interrupt alveolar recruitment maneuver and reasons (heart rate > 150bpm or < 60bpm; reduction of mean blood pressure to < 65mmHg or systolic blood pressure < 90mmHg; reduction of SpO_2_ < 88% for > 30 seconds; severe arrhythmia: acute atrial fibrillation or flutter, ventricular tachycardia); (2) hypotension (mean blood pressure < 65mmHg) within one hour after randomization; (3) use of vasopressors (norepinephrine or dopamine) within one hour; (4) hypotension or need for vasopressors within one hour; (5) hypoxemia (PaO_2_ < 55mmHg) within one hour; (6) severe acidosis (pH < 7.10) within one hour; (7) pneumothorax requiring drainage in the first 7 days after randomization; and (8) any barotrauma in the first 7 days after randomization. The Coordinating Centre also sent reports of serious study-related adverse events to the DMC immediately after receiving them.

### Sample size

ART is an event-driven study designed to last until 520 events (death within 28 days) are observed. This number of events is sufficient to detect a hazard ratio of 0.75 (i.e., relative reduction in event rate of 25%), considering a type I error of 5%, 90% power, and similar allocation of subjects to each group.

An important advantage of using an event-driven strategy is that it ensures adequate power for the study as well as recruitment of an adequate number of patients - if the event rate turns out to be higher than that reported in the literature, the study will be completed with a smaller sample size than would be required by a method based on the total sample size; consequently, there is no unnecessary inclusion of patients. If the event rate turns out to be lower than that reported in the literature, the study is not interrupted before it has adequate power, as might be the case if the total sample size method was used.

### Statistical analysis

All statistical analyses will be conducted according to the intention-to-treat principle. Thus, patients will be analyzed according to the arm to which they were allocated (ART or ARDSNet).

Continuous distribution of the data will be assessed by visual inspection of histograms and D'Agostino-Pearson's normality tests. For the experimental and control arms, the baseline characteristics will be expressed as counts and percentages, means and standard deviations (SD), or medians and interquartile ranges (IQR) whenever appropriate as indicated in mock tables 2 to 6, which we intend to include in the main results paper.

**Table 2:** Baseline characteristics of the patients

Characteristic	ART	ARDSNet
Age (years)	xx.x ± xx.x	xx.x ± xx.x
Female sex, N/total N (%)	x/x (xx.x)	x/x (xx.x)
SAPS3 score	xx.x ± xx.x	xx.x ± xx.x
No. of non-pulmonary organ failures	xx.x ± xx.x	xx.x ± xx.x
Septic shock, N/total N (%)	x/x (xx.x)	x/x (xx.x)
Cause of ARDS		
Pulmonary ARDS, N/total N (%)	x/x (xx.x)	x/x (xx.x)
Pneumonia	x/x (xx.x)	x/x (xx.x)
Gastric aspiration	x/x (xx.x)	x/x (xx.x)
Lung contusion	x/x (xx.x)	x/x (xx.x)
Near drowning	x/x (xx.x)	x/x (xx.x)
Extrapulmonary ARDS, N/total N (%)	x/x (xx.x)	x/x (xx.x)
Non-septic shock	x/x (xx.x)	x/x (xx.x)
Sepsis/septic shock	x/x (xx.x)	x/x (xx.x)
Trauma without lung contusion	x/x (xx.x)	x/x (xx.x)
Cardiac surgery	x/x (xx.x)	x/x (xx.x)
Other major surgery	x/x (xx.x)	x/x (xx.x)
Head trauma	x/x (xx.x)	x/x (xx.x)
Smoke inhalation	x/x (xx.x)	x/x (xx.x)
Multiple transfusions	x/x (xx.x)	x/x (xx.x)
Drug or alcohol abuse	x/x (xx.x)	x/x (xx.x)
Other	x/x (xx.x)	x/x (xx.x)
Prone position, N/total N (%)	x/x (xx.x)	x/x (xx.x)
Time since onset of ARDS (hours)	x/x (xx.x)	x/x (xx.x)
Days intubated prior to randomization, median (IQR)	x/x (xx.x)	x/x (xx.x)
Respiratory measures		
PaO_2_ at FIO_2_=1	xx.x ± xx.x	xx.x ± xx.x
Tidal volume (mL/kg predicted body weight)	xx.x ± xx.x	xx.x ± xx.x
Plateau airway pressure (cmH_2_O)	xx.x ± xx.x	xx.x ± xx.x
Minute ventilation (L/min)	xx.x ± xx.x	xx.x ± xx.x
Respiratory rate (breaths/min)	xx.x ± xx.x	xx.x ± xx.x
Driving pressure	xx.x ± xx.x	xx.x ± xx.x
Positive end-expiratory pressure (cmH_2_O)	xx.x ± xx.x	xx.x ± xx.x
Respiratory system static compliance (mL/cmH_2_O)	xx.x ± xx.x	xx.x ± xx.x

ART - Alveolar Recruitment Trial; SAPS - Simplified Acute Physiology Score; ARDS - acute respiratory distress syndrome; PaO_2_ - partial pressure of arterial oxygen; FIO_2_ - fraction of inspired oxygen. Plus-minus values are the means ± standard deviation.

**Table 6:** Outcomes

Outcome	ART	ARDSNet	Hazard ratio (95%CI)	p value
Primary outcome				
Death within 28 days, N events/N total (%)	x/x (xx.x)	x/x (xx.x)	x.xx (x.xx - x.xx)	x.xx
Secondary outcomes				
Death in hospital, N events/N total (%)*	x/x (xx.x)	x/x (xx.x)	x.xx (x.xx - x.xx)*	x.xx
Death in intensive care unit, N events/N total (%)*	x/x (xx.x)	x/x (xx.x)	x.xx (x.xx - x.xx)*	x.xx
Death within 6 months, N events/N total (%)	x/x (xx.x)	x/x (xx.x)	x.xx (x.xx - x.xx)	x.xx
Length of intensive care unit stay (days)†	xx.x ± xx.x	xx.x ± xx.x	x.xx (x.xx - x.xx)†	x.xx
median (IQR)	xx (xx to xx)	xx (xx to xx)		
Length of hospital stay (days)†	xx.x ± xx.x	xx.x ± xx.x	x.xx (x.xx - x.xx)†	x.xx
median (IQR)	xx (xx to xx)	xx (xx to xx)		
No. of ventilator-free days from day 1 to day 28†	xx.x ± xx.x	xx.x ± xx.x	x.xx (x.xx - x.xx)†	x.xx
median (IQR)	xx (xx to xx)	xx (xx to xx)		
Pneumothorax requiring drainage within 7 days, N events/N total (%)*	x/x (xx.x)	x/x (xx.x)	x.xx (x.xx - x.xx)*	x.xx
Barotrauma within 7 days, N events/N total (%)†*	x/x (xx.x)	x/x (xx.x)	x.xx (x.xx - x.xx)*	x.xx
Exploratory outcomes				
Death with refractory hypoxemia within 7 days, N events/N total (%)*	x/x (xx.x)	x/x (xx.x)	x.xx (x.xx - x.xx)*	x.xx
Death with refractory acidosis within 7 days, N events/N total (%)*	x/x (xx.x)	x/x (xx.x)	x.xx (x.xx - x.xx)*	x.xx
Death with barotrauma within 7 days, N events/N total (%)*	x/x (xx.x)	x/x (xx.x)	x.xx (x.xx - x.xx)*	x.xx
Cardiorespiratory arrest on day 1, N events/N total (%)*	x/x (xx.x)	x/x (xx.x)	x.xx (x.xx - x.xx)*	x.xx
Need for commencement/increase of vasopressors or hypotension (MAP < 65mmHg) within 1 hour*	x/x (xx.x)	x/x (xx.x)	x.xx (x.xx - x.xx)*	x.xx
Refractory hypoxemia (PaO_2_ < 55mmHg) within 1 hour, N (%)*	x/x (xx.x)	x/x (xx.x)	x.xx (x.xx - x.xx)*	x.xx
Severe acidosis (pH < 7.10) within 1 hour, N (%)*	x/x (xx.x)	x/x (xx.x)	x.xx (x.xx - x.xx)*	x.xx

ART - Alveolar Recruitment Trial; 95%CI - 95% confidence interval; MAP - mean arterial pressure; PaO_2_ - partial pressure of arterial oxygen.

* Effect estimates are the risk ratios.

† Effect estimates are the mean difference.

Hypothesis tests will be two-sided with a significance level of 5%. We will not adjust p values for multiple comparisons. Analyses will be performed using the R (R Core Team, 2016, Vienna, Austria) program.

### Trial profile

**Figura 2:**
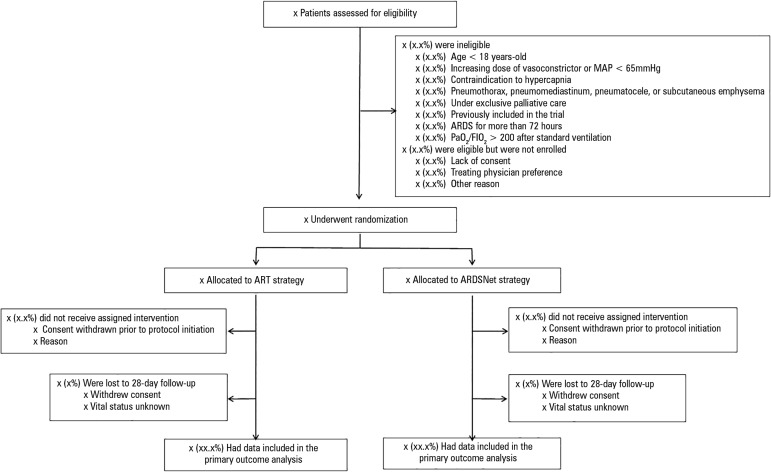
Study flow. MAP - mean arterial pressure; ARDS - acute respiratory distress syndrome; PaO_2_ - partial pressure of arterial oxygen; FIO_2_ - fraction of inspired oxygen; ART - Alveolar Recruitment for ARDS Trial.

Patient flow will be presented as a Consolidated Standards of Reporting Trials diagram (Figure 2).

### Baseline comparisons

We will present patients' baseline characteristics by study arm, as depicted in table 2.

### Adherence to study interventions, respiratory variables

We will report data to assess adherence to the components of the recruitment maneuver and PEEP titration procedure, as shown in table 3, as well as respiratory variables from hour 1 to day 7 for both arms, as shown in table 4. Fluid balance, weight gain and co-interventions during the first seven days of treatment will also be presented, as depicted in table 5.

**Table 3:** Maximum alveolar recruitment maneuver and titrated PEEP levels

Characteristic	ART
Maximum alveolar recruitment maneuver, N (%)	
Completed (PEEP = 45cmH_2_O)	x/x (x.x)
Completed (PEEP = 35cmH_2_O)	x/x (x.x)
Interrupted at PEEP = 45cmH_2_O	x/x (x.x)
Interrupted at PEEP = 35cmH_2_O	x/x (x.x)
Interrupted at PEEP = 30cmH_2_O	x/x (x.x)
Interrupted at PEEP = 25cmH_2_O	x/x (x.x)
Interrupted at other PEEP levels	x/x (x.x)
Not attempted	x/x (x.x)
Neuromuscular blocking agent immediately before alveolar recruitment maneuver, N (%)	x/x (x.x)
Volemia optimized before alveolar recruitment maneuver, N (%)*	x/x (x.x)
Reason for interrupting alveolar recruitment maneuver, N (%)	
Heart rate < 60bpm or > 150bpm	x/x (x.x)
Mean blood pressure < 65mmHg or systolic blood pressure < 90mmHg	x/x (x.x)
SpO_2_ < 88% for longer than 30s	x/x (x.x)
Other	x/x (x.x)
Titrated PEEP (cmH_2_O)	xx.x ± xx.x
Alveolar recruitment maneuver repeated immediately after PEEP titration, N (%)	x/x (x.x)
Recruitment maneuver repeated on days 1 to 7, N (%)	
No	x/x (x.x)
Once	x/x (x.x)
Twice	x/x (x.x)
Three or more times	x/x (x.x)

ART - Alveolar Recruitment Trial; PEEP - positive end-expiratory pressure; SpO_2_ - peripheral oxygen saturation.

* Volemia is considered optimized when fluids are administered before recruitment maneuver if dynamic signs of fluid responsiveness are present (such as pulse pressure variation >13%) or central venous pressure < 10cmH_2_O. Plus-minus values are the means ± standard deviation.

**Table 4:** Respiratory variables during the first seven days of treatment

Variable	1 hour			Day 1			Day 3			Day 7		
	ART	ARDSNet	p value	ART	ARDSNet	p value	ART	ARDSNet	p value	ART	ARDSNet	p value
Tidal volume (mL/kg of predicted body weight)	x.x ± x.x	x.x ± x.x	x.xx	x.x ± x.x	x.x ± x.x	x.xx	x.x ± x.x	x.x ± x.x	x.xx	x.x ± x.x	x.x ± x.x	x.xx
Tidal volume > 6.5mL/kg of predicted body weight, N/total N (%)	x/x (x.x)	x/x (x.x)	x.xx	x/x (x.x)	x/x (x.x)	x.xx	x/x (x.x)	x/x (x.x)	x.xx	x/x (x.x)	x/x (x.x)	x.xx
PEEP (cmH_2_O)	x.x ± x.x	x.x ± x.x	x.xx	x.x ± x.x	x.x ± x.x	x.xx	x.x ± x.x	x.x ± x.x	x.xx	x.x ± x.x	x.x ± x.x	x.xx
Plateau pressure (cmH_2_O)	x.x ± x.x	x.x ± x.x	x.xx	x.x ± x.x	x.x ± x.x	x.xx	x.x ± x.x	x.x ± x.x	x.xx	x.x ± x.x	x.x ± x.x	x.xx
Plateau pressure > 30cmH_2_O, N/total N (%)	x/x (x.x)	x/x (x.x)	x.xx	x/x (x.x)	x/x (x.x)	x.xx	x/x (x.x)	x/x (x.x)	x.xx	x/x (x.x)	x/x (x.x)	x.xx
Driving pressure (cmH_2_O)	x.x ± x.x	x.x ± x.x	x.xx	x.x ± x.x	x.x ± x.x	x.xx	x.x ± x.x	x.x ± x.x	x.xx	x.x ± x.x	x.x ± x.x	x.xx
Respiratory system static compliance (mL/cmH_2_O)	x.x ± x.x	x.x ± x.x	x.xx	x.x ± x.x	x.x ± x.x	x.xx	x.x ± x.x	x.x ± x.x	x.xx	x.x ± x.x	x.x ± x.x	x.xx
Respiratory rate (breaths/min)	x.x ± x.x	x.x ± x.x	x.xx	x.x ± x.x	x.x ± x.x	x.xx	x.x ± x.x	x.x ± x.x	x.xx	x.x ± x.x	x.x ± x.x	x.xx
PaO_2_/FIO_2_	x.x ± x.x	x.x ± x.x	x.xx	x.x ± x.x	x.x ± x.x	x.xx	x.x ± x.x	x.x ± x.x	x.xx	x.x ± x.x	x.x ± x.x	x.xx
PaCO_2_ (mmHg)	x.x ± x.x	x.x ± x.x	x.xx	x.x ± x.x	x.x ± x.x	x.xx	x.x ± x.x	x.x ± x.x	x.xx	x.x ± x.x	x.x ± x.x	x.xx
Arterial pH	x.x ± x.x	x.x ± x.x	x.xx	x.x ± x.x	x.x ± x.x	x.xx	x.x ± x.x	x.x ± x.x	x.xx	x.x ± x.x	x.x ± x.x	x.xx

ART - Alveolar Recruitment Trial; PEEP - positive end-expiratory pressure; PaO_2_ - partial pressure of arterial oxygen; PaCO_2_ - partial pressure of carbon dioxide Plus-minus values are the means ± standard deviation.

**Table 5:** Fluid balance, weight gain and co-interventions during the first seven days of treatment

	ARDSNET (n=x)	ART (n=x)	p value
24 hours fluid balance (mL)			
Day 1	xx.x ± xx.x	xx.x±xx.x	x.xx
Day 3	xx.x ± xx.x	xx.x±xx.x	x.xx
Weight gain (kg)			
Baseline to day 1	xx.x ± xx.x	xx.x±xx.x	x.xx
Baseline to day 3	xx.x ± xx.x	xx.x±xx.x	x.xx
Baseline to day 7	xx.x ± xx.x	xx.x±xx.x	x.xx
Use of vasopressors, N/total N (%)	x/x (xx.x)	x/x (xx.x)	x.xx
Days of vasopressor use, median (IQR)	median (IQR)	median (IQR)	x.xx
Neuromuscular blockade, N/total N (%)	x/x (xx.x)	x/x (xx.x)	x.xx
Days of neuromuscular blocker use, median (IQR)	median (IQR)	median (IQR)	x.xx
Sedative infusion, N/total N (%)	x/x (xx.x)	x/x (xx.x)	x.xx
Days of sedative infusion, median (IQR)	median (IQR)	median (IQR)	x.xx
Narcotic infusion, N/total N (%)	x/x (xx.x)	x/x (xx.x)	x.xx
Days of narcotic infusion, median (IQR)	median (IQR)	median (IQR)	x.xx
Use of corticosteroid, N/total N (%)	x/x (xx.x)	x/x (xx.x)	x.xx
Days of corticosteroid, median (IQR)	median (IQR)	median (IQR)	x.xx
Rescue therapies, N/total N (%)	x/x (xx.x)	x/x (xx.x)	x.xx
Prone position, N/total N (%)	x/x (xx.x)	x/x (xx.x)	x.xx
Inhaled nitric oxide, N/total N (%)	x/x (xx.x)	x/x (xx.x)	x.xx
High frequency oscillation, N/total N (%)	x/x (xx.x)	x/x (xx.x)	x.xx
Extracorporeal membrane oxygenation, N/total N (%)	x/x (xx.x)	x/x (xx.x)	x.xx

ART - Alveolar Recruitment for ARDS Trial.

### Effect on outcomes

We will report the number and percentage of deaths within 28 days after randomization (Table 6). Survival within 28 days in both groups will be assessed using Kaplan-Meier curves, and hazard ratios with a 95% confidence interval will be calculated with Cox proportional hazard models without adjustment for other co-variates.

The two-sided α-level for the primary outcome final analysis is 0.042 to account for alpha from the two interim analyses with boundaries at one-sided α = 0.01.

We will extend the survival analysis until the 6-month follow-up and present the results using Kaplan-Meier curves and the hazard ratio with a 95% confidence interval, which will be calculated with Cox proportional hazard models. We will also test proportional hazard assumptions and propose alternative parametric survival models if the proportionality assumption is not sustained.^(11)^

We will assess the effect of the intervention on ICU and in-hospital mortality with risk ratios and 95% confidence intervals calculated with Wald's likelihood ratio approximation test and with chi-squared tests for hypothesis testing. The effects of the intervention on length of hospitalization, ICU stay and ventilator-free days (until 28^th^ day since randomization) will be estimated with generalized linear models considering distributions that will fit a possible heavy right-tailed distribution (such as *gamma*, inverse *Gaussian*, or truncated *Poisson* for ventilator-free days specifically), choosing the best fit according to the model's deviance.^(12)^

We will also address the effect of the intervention on the secondary safety outcomes described in mock table 6. Every comparison will be assessed by risk ratios with the respective 95%CI calculated according to Wald's likelihood ratio approximation test.

### Subgroup analyses

Treatment effects on 28-day mortality will be analyzed in the following subgroups: (1) PaO_2_/FIO_2_ ≤ 100 *versus* >100mmHg; (2) Simplified Acute Physiology Score (SAPS) 3 score <50 *versus* ≥ 50; (3) pulmonary ARDS *versus* extrapulmonary ARDS; (4) time of ARDS ≤ 36 hours *versus* > 36 to < 72 hours; (5) mechanical ventilation ≤ 2 days; 3 to 4 days; ≥ 5 days; and (6) prone position. Subgroups will be classified according to data obtained at baseline, except for prone position, which will be classified according to the position (prone or not prone) determined 1 hour after randomization. The reason for considering 1-hour data for determining prone *versus* other positions is because we have recommended to investigators that patients with an indication for prone positioning should be moved to that position immediately after randomization. The effects on subgroups will be evaluated according to the interaction effects between each subgroup and the study arms by Cox proportional hazard models.

### Other exploratory analyses

We will test whether the effects of the intervention on the primary and secondary outcomes are similar before and after the protocol amendment of June 2015.

As a sensitivity analysis, we will estimate the effect of the study intervention on the primary outcome using Cox proportional hazard models with adjustment for the following covariates determined at baseline: age, SAPS 3 score, and PaO_2_/FIO_2_.

Finally, if there is evidence that the experimental treatment decreases 28-day mortality, we should assess whether the driving pressure mediates the eventual effects of the randomly assigned treatment on 28-day mortality. Mediators are variables that are affected by treatment-group assignment and that subsequently affect the outcome.^(13)^ Therefore, mediators are on the causal pathway of the relation between treatment and outcome, at least partly explaining the effects of the treatment on the outcome. In a first step, we plan to assess the effect of the driving pressure determined on day 1 on 28-day mortality. This exploratory analysis will be conducted using a Cox proportional hazard model adjusted for treatment assignment (ART or ARDSNet), age, SAPS 3 score, and baseline PaO_2_/FIO_2_. The effects of other respiratory variables determined on day 1 (tidal volume, PEEP, plateau pressure, static compliance of the respiratory system) on 28-day mortality will also be modeled by adding them to the previously described Cox proportional hazard model.

In a second step, we will use the bootstrapping technique to test the mediation models, an alternative to Baron and Kenny's causal steps model technique to evaluate mediation.^(14)^ We will use the R package *mediation*.^(15)^ These models will be adjusted for the baseline tidal elastance of the respiratory system to avoid possible confounding due to differences in the severity of the underlying respiratory illness. The outputs of the mediation models will be the average causal mediation effect (indirect effect) and direct effect. The indirect effect expresses the proportion of the treatment effect occurring via the mediator, and the direct effect expresses the proportion of the treatment effect that is independent of the mediator.

### Missing data

We anticipate no or minimal losses to follow-up for the primary and secondary outcome data. We plan to carry out complete-case analyses for the primary and secondary outcomes, that is, we will exclude patients with missing data. However, if we end the trial with a loss of primary outcome data for 1% or more of patients, we will carry out a sensitivity analysis using multiple imputation techniques.

## CONCLUSION

According to the best trial practice, we report our statistical analysis plan and data management plan prior to locking the database and starting analyses. We anticipate that this document will prevent analysis bias and enhance the utility of the reported results.

## ACKNOWLEDGMENTS

Funded by the *Programa de Apoio ao Desenvolvimento Institucional* of the *Sistema* Único *de Saúde* (PROADI-SUS) from the Brazilian Ministry of Health.
